# Identification of a Two-Gene Signature and Establishment of a Prognostic Nomogram Predicting Overall Survival in Diffuse-Type Gastric Cancer

**DOI:** 10.3390/curroncol30010014

**Published:** 2022-12-23

**Authors:** Songyao Chen, Jiannan Xu, Songcheng Yin, Huabin Wang, Guangyao Liu, Xinghan Jin, Junchang Zhang, Huijin Wang, Han Wang, Huan Li, Jianming Liang, Yulong He, Changhua Zhang

**Affiliations:** 1 Digestive Medicine Center, The Seventh Affiliated Hospital of Sun Yat-sen University, Shenzhen 518107, China; 2 Department of Thoracic Surgery, The Third Affiliated Hospital of Sun Yat-sen University, Guangzhou 510630, China; 3 Division of Hematology/Oncology, Department of Pediatrics, The Seventh Affiliated Hospital of Sun Yat-sen University, Shenzhen 518107, China; 4 Gastrointestinal Surgery Center, The First Affiliated Hospital of Sun Yat-sen University, Guangzhou 510080, China

**Keywords:** diffuse-type gastric cancer, prognosis, nomogram, gene signature

## Abstract

Background: It is widely acknowledged that the molecular biological characteristics of diffuse-type gastric cancer are different from intestinal-type gastric cancer. Notwithstanding that significant progress in high-throughput sequencing technology has been made, there is a paucity of effective prognostic biomarkers for diffuse gastric cancer for clinical practice. Methods: We downloaded four GEO datasets (GSE22377, GSE38749, GSE47007 and GSE62254) to establish and validate a prognostic two-gene signature for diffuse gastric cancer. The TGCA-STAD dataset was used for external validation. The optimal gene signature was established by using Cox regression analysis. Receiver operating characteristic (ROC) methodology was used to find the best prognostic model. Gene set enrichment analysis was used to analyze the possible signaling pathways of the two genes (MEF2C and TRIM15). Results: A total of four differently expressed genes (DEGs) (two upregulated and two downregulated) were identified. After a comprehensive analysis, two DEGs (MEF2C and TRIM15) were utilized to construct a prognostic model. A prognostic prediction model was constructed according to T stage, N stage, M stage and the expression of MEF2C and TRIM15. The area under the time-dependent receiver operator characteristic was used to evaluate the performance of the prognosis model in the GSE62254 dataset. Conclusions: We demonstrated that MEF2C and TRIM15 might be key genes. We also established a prognostic nomogram based on the two-gene signature that yielded a good performance for predicting overall survival in diffuse-type gastric cancer.

## 1. Introduction

Gastric cancer (GC) is the third most common cause of cancer-related death globally, with a low 5-year survival rate [1]. Indeed, in recent years, with the establishment of multidisciplinary team (MDT) care, GC treatment has substantially improved. Albeit multiple therapeutic approaches are available currently, it is difficult to accurately determine the optimal treatment for an individual gastric cancer (GC) patient due to clinical and genetic heterogeneity [2]. The Lauren classification is an internationally recognized histopathological classification that sorts GC into three subtypes: intestinal type, diffuse type and mixed type [3]. However, several studies have shown significant heterogeneity in biological behavior among the three subtypes of gastric cancers [4,5,6,7]. Importantly, diffuse-type GC has been documented to exhibit more aggressive behavior and a poorer prognosis, accounting for one-third of all GC patients [5]. This subtype of GC is more common in women and younger patients and is more genetic-related [8]. Nowadays, the AJCC TNM staging system is widely used to evaluate the prognosis of GC patients [9,10]. Nevertheless, multiple studies reported that the AJCC TNM staging system sometimes exhibited poor accuracy in predicting the prognosis of cancer patients, partly due to staging migration [11]. With the advancement of cancer molecular biology, cancer-specific gene signatures have been harnessed to develop new prediction tools. However, there are limited prognosis-related genes associated with diffuse-type GC. Accordingly, an effective prediction model is urgently needed to help oncologists evaluate GC patient prognosis in the clinic.

In this study, we obtained three GC datasets with Lauren classification data from the GEO database to identify differentially expressed genes (DEGs). Then, the DEGs were validated with external datasets. Univariate regression analysis and Kaplan–Meier analysis were applied to select the overall survival-related DEGs. A prognostic nomogram incorporating prognostic gene signatures and clinical survival-related characteristics was established to predict overall survival. In summary, we uncovered new gene signatures for diffuse-type GC and established a nomogram that exhibited good performance in predicting the overall survival of diffuse-type GC patients.

## 2. Materials and Methods

### 2.1. Data Collection and Processing

The gene expression profile data (GSE22377, GSE38749, GSE47007 and GSE62254) were searched and downloaded from the Gene Expression Omnibus (GEO) (https://www.ncbi.nlm.nih.gov/geo/ (accessed on 1 June 2022)) database. All included datasets met the following criteria: (1) histopathological information with detailed records of the Lauren classification available; (2) more than ten samples included; (3) gene expression profile in RNA level; (4) datasets were described in English language. Exclusion criteria included not diffuse- and intestinal-type gastric cancer; normal tissue of diffuse-type gastric cancer patients. In addition, data of 83 diffuse-type gastric cancer patients were extracted from TCGA-STAD dataset (https://portal.gdc.cancer.gov (accessed on 1 June 2022)) for external validation.

Probes were matched to the gene symbols using the annotation files provided by the manufacturer. The median expression value was calculated if multiple probes matched a single gene. Robust multi-array average (RMA)-normalized data were log2-transformed for further analysis. Limma package [12] in R/Bioconductor software (version 3.6.1, R Foundation for Statistical Computing, Vienna, Austria) was applied to screen the DEGs between diffuse-type GC and intestinal-type GC in GSE22377, GSE38749 and GSE47007 datasets. | log2Fold change (FC) | ≥ 1 and *p* value < 0.05 were set as the thresholds for significant differential expression.

### 2.2. Validation of Expression Level of DEGs

The screened DEGs were validated with a large dataset (GSE62254). Diffuse-type and intestinal-type histopathological data were available from the dataset. We analyzed the expression of DEGs between these two subtypes. Finally, a boxplot was constructed to visualize the expression level.

### 2.3. Kaplan–Meier Plotter Analysis

Kaplan–Meier Plotter (https://kmplot.com/analysis/ (accessed on 1 June 2022)) is an online website that integrates RNA-sequencing data of GEO dataset and survival information [13]. We used Gastric Cancer section of Kaplan–Meier Plotter (https://kmplot.com/analysis/index.php?p=service&cancer=gastric (accessed on 1 June 2022)) to explore the prognostic value of DEGs in diffuse-type gastric cancer [14].

### 2.4. Clinical Correlation Analysis and Biological Process Prediction

We extracted the clinical information from the GSE62254 dataset. SPSS 24.0 was used to perform a Chi-square test between every single gene and clinical pathological characteristics. A *p* value less than 0.05 was considered statistically significant. To understand the biological process of the identified prognostic gene signatures, gene set enrichment analysis (GSEA) was performed by using a Java GSEA desktop application (downloaded from http://www.broad.mit.edu/gsea (accessed on 1 June 2022)). The GSE62254 samples were divided into high- and low-expression groups according to the median value. The GSE62254 dataset was analyzed with the GTM file (c2.KEGG.v6.2) to identify enriched KEGG pathways. Four files containing expression datasets, gene sets, phenotype labels and chip platforms were required for running GSEA. |NES| > 1 and FDR < 0.25 were considered statistically significant. 

### 2.5. The Establishment of the Predictive Nomogram

After testing for collinearity, prognostic gene signatures and relevant clinical parameters were included to establish a prognostic nomogram via a stepwise Cox regression model to predict the 1-, 3- and 5-year overall survival of diffuse-type gastric cancer patients in the GSE62254 dataset. A time-dependent ROC curve, Harrell’s concordance index and a calibration curve were utilized to assess the performance of the prognostic nomogram. Decision curve analysis was used to evaluate the net benefit of the program compared with TNM staging alone.

Based on the prognostic nomogram, the diffuse-type gastric cancer samples from the GSE62254 dataset were assigned to high-risk and low-risk score groups according to the median risk score. Kaplan–Meier analysis was performed to demonstrate the relationship between risk score and overall survival time by using the “survival” package. A log-rank test was used to distinguish the differences between groups.

### 2.6. External Validation of Two-Gene Signature-Based Nomogram by TCGA Dataset

To further confirm the prediction value of the two-gene signature nomogram, we performed ROC analysis to show the predictive performance of TNM staging and the nomogram-based model. Kaplan–Meier analysis and the log-rank test were applied to demonstrate the survival difference between the high-risk group and low-risk group. Decision curve analysis was also utilized to quantify the clinical benefits of the nomogram at different threshold probabilities. The above analyses of external validation were performed by using the TCGA-STAD dataset.

## 3. Results

### 3.1. Identification of Diffuse-Type Gastric Cancer-Specific Gene Signatures

**Figure 1:**
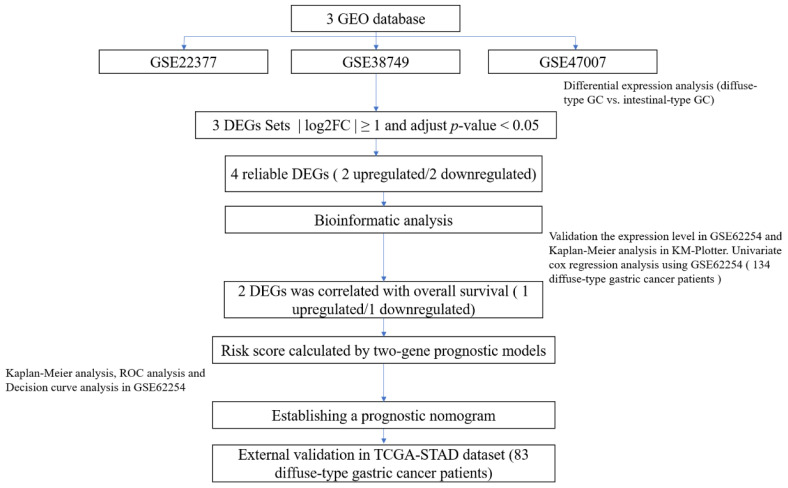
Flowchart showing the process of establishing the gene signature and prognostic nomogram of diffuse-type gastric cancer in this study.

The flowchart of the screening process used in our study to identify diffuse-type gastric cancer gene signatures is shown in Figure 1. The details of the GEO datasets included in this study are displayed in Table 1. A total of 991 (GSE22377), 166 (GSE38749) and 171 (GSE47007) DEGs were identified between the diffuse-type and intestinal-type gastric cancer datasets. Two genes (COL4A3, MEF2C) were highly expressed in diffuse-type gastric cancer, whereas two (TRIM15, MMP12) were lowly expressed in diffuse-type gastric cancer.

### 3.2. Validation of the Expression Level of Four Differentiated Expressed Genes

**Figure 2:**
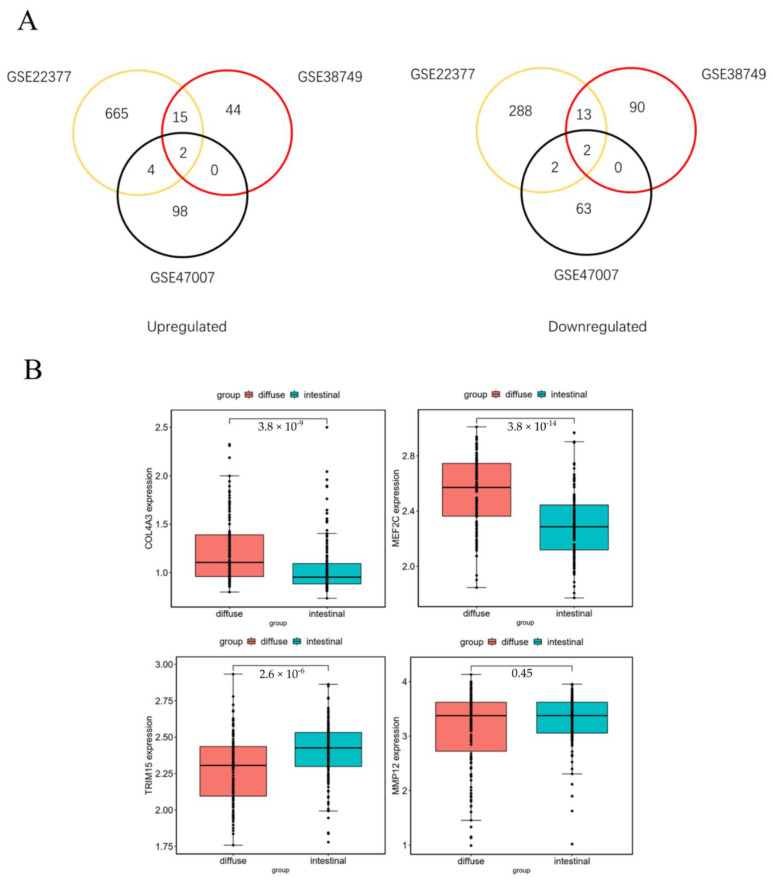
(**A**) The Venn diagram shows the DEGs with upregulated genes and downregulated genes in diffuse-type GC. (**B**) Expression levels of four DEGs between diffuse-type and intestinal-type gastric cancer were validated in GSE62254 dataset.

In this study, the expression level of the four DEGs identified was validated in a large dataset (GSE62254). Two upregulated and one downregulated gene were identified (Figure 2B).

### 3.3. Clinical Correlation Analysis of Three DEGs

Detailed clinical information of 134 patients from the GSE62254 dataset was extracted. A Chi-square test was used to evaluate the relationship between the three DEGs and clinical pathological characteristics (Table 2). In brief, the expression level of COL4A3 and TRIM15 were significantly correlated with the T stage and age, respectively, while the expression level of MEF2C was significantly correlated with age, T stage and TNM stage.

### 3.4. Kaplan–Meier Analysis and Evaluation of Prognostic Factors in Diffuse-Type Gastric Cancer

**Figure 3:**
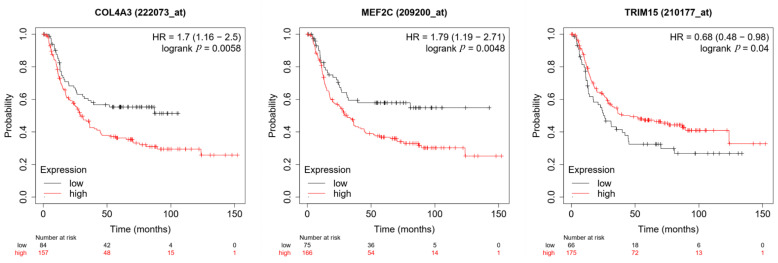
Validation of prognostic value of three genes in diffuse-type gastric cancer (n = 241) by Kaplan–Meier Plotter.

The survival information of COL4A3, MEF2C and TRIM15 was freely obtained in Kaplan–Meier Plotter. In this study, we assessed the difference between the expression level of the three DEGs and overall survival in diffuse-type gastric cancer. Notably, in the results of Kaplan–Meier Plotter, we used the best cutoff value of COL4A3, MEF2C and TRIM15 expression to divide diffuse-type gastric cancer patients into a high expression- and low expression-group, respectively. It was found that the high expression of COL4A3 and MEF2C and the low expression of TRIM15 were associated with worse OS for diffuse-type gastric cancer patients (Figure 3). However, the false discovery rate (FDR) was 50%, 50% and over 50% for the survival difference of COL4A3, MEF2C and TRIM15, respectively. These FDR values were high. Thus, it made it difficult for us to evaluate the prognostic value of these genes.

A univariate Cox regression analysis was performed to evaluate the prognostic value and identify the risk factors in diffuse-type gastric cancer. The results of the univariate Cox regression analysis demonstrated that T stage (*p* < 0.01), N stage (*p* < 0.01), M stage (*p* < 0.001) and the expression level of MEF2C (*p* < 0.01) and TRIM15 (*p* < 0.05) were significantly correlated with overall survival in diffuse-type gastric cancer (Table 3).

### 3.5. Establishment of the Prognostic Nomogram of Diffuse-Type Gastric Cancer

**Figure 4:**
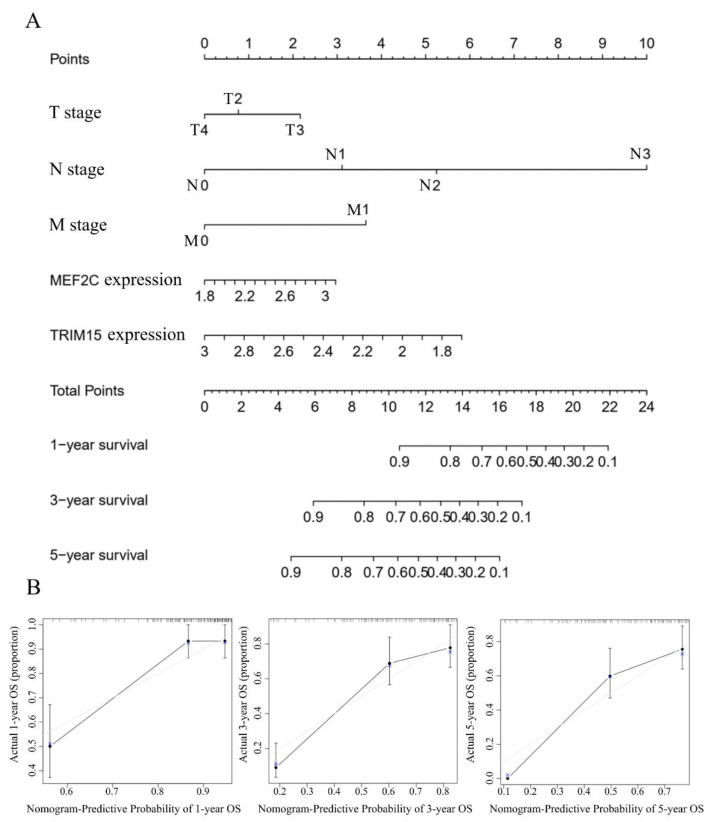
Construction and internal validation of nomogram. (**A**) The predictive nomogram was established with T-stage, N-stage, M-stage and expression level of MEF2C and TRIM15 in diffuse-type gastric cancer. (**B**) A comparison of predictive and actual outcome for 1-, 3- and 5-year survival probabilities in the nomogram is demonstrated in calibration curves for internal validation.

The clinical information of the 134 diffuse-type gastric cancer patients from the GSE62254 dataset was used to construct a prognostic nomogram for predicting 1-, 3-, 5-year overall survival based on a stepwise Cox regression model (Figure 4A). T stage, N stage, M stage and the expression of TRIM15 and MEF2C were parameters included in the nomogram. The calibration curves showed a good consistency between the actual and the nomogram-predicted 1-, 3- and 5-year overall survival probabilities (Figure 4B). The risk score was calculated as Formula (1):

e^((−0.52425 + [0.621×Expression value of MEF2C] + [(−1.2188)×Expression value of TRIM15] + β_T_ + β_N_ + β_M_)) (1)

When T stage is T2, T3 or T4, the value of β_T_ is 0, 0.3812 or −0.2084, respectively.

When N stage is N0, N1, N2 or N3, the value of β_N_ is 0, 0.8741, 1.4293 or 2.7244, respectively.

When M stage is M0 or M1, the value of β_M_ is 0 or 0.994, respectively.

### 3.6. Evaluation the Predictive Performance of Nomogram and External Validation of Nomogram

**Figure 5:**
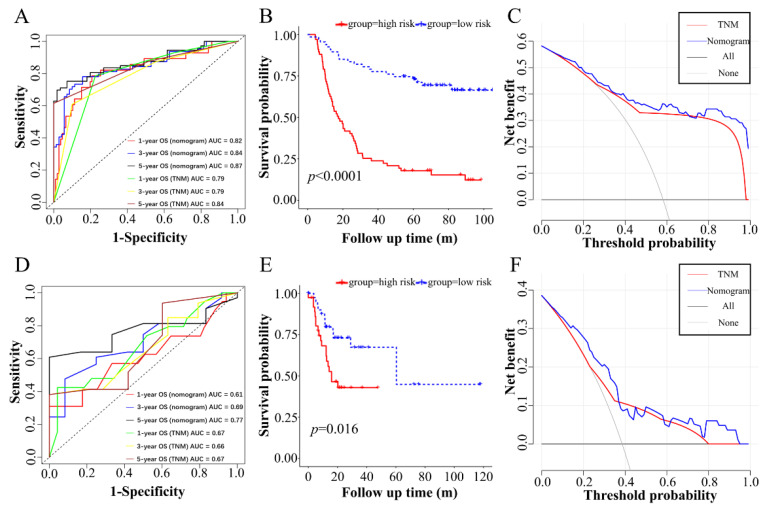
(**A**) Receiver operating characteristic (ROC) curves were used to compare the predictive ability of nomogram model and TNM stage for 1-, 3- and 5-year survival probabilities in training set. (**B**) The Kaplan–Meier curve shows the high-risk group with worse prognosis in training set. (**C**) Decision curve analysis of nomogram in training set for OS. (**D**) The ROC curves of nomogram in external validation set (TCGA-STAD). (**E**) Kaplan–Meier analysis of high-risk group and low-risk group in external validation set (TCGA-STAD). (**F**) Decision curve analysis of nomogram in external validation set.

The AUC of the predicted 1-, 3- and 5-year overall survival were 0.82, 0.84 and 0.87, respectively (Figure 5A). When the seventh AJCC TNM stage was used, the AUC values for the 1-, 3- and 5-year overall survival predictions were 0.79, 0.79 and 0.84, respectively (Figure 5A). As seen in Figure 4A, the calculated overall score could estimate the survival prognosis (1-, 3- and 5-year survival probabilities), and the C-index of our nomogram model was 0.766 (95% CI = 0.711 ~ 0.821). The Kaplan–Meier analysis showed a significant difference in prognostic value between the high-risk and low-risk groups (Figure 5B). Decision curve analysis showed the effectiveness of the nomogram was better than the seventh AJCC TNM staging system.

For external validation, using the TCGA-STAD dataset, the results of ROC curves, Kaplan–Meier analysis and decision curve analysis were similar with the training set (GSE62254). The AUC of predicted 1-, 3- and 5-year overall survival were 0.61, 0.69 and 0.77, which were better than the TNM stating system (Figure 5D) in the TCGA-STAD dataset. Also, the patients in the low-risk group had a more favorable overall prognosis than the high-risk group in the validation set (Figure 5E). Similarly, decision curve analysis also demonstrated the net benefit of the nomogram was better than the TNM staging system in the TCGA-STAD dataset (Figure 5F). Therefore, the nomogram showed better discriminatory ability than the seventh AJCC TNM classification.

### 3.7. Scatter Point

**Figure 6:**
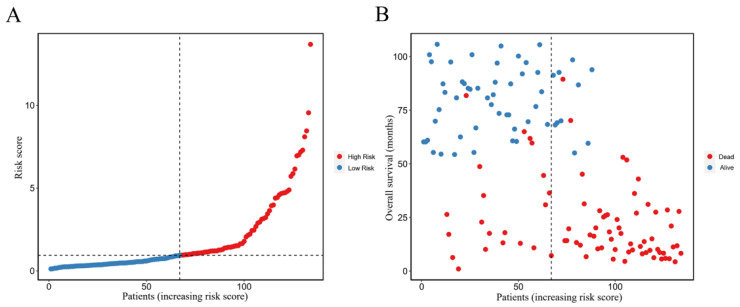
The association between risk score, OS and survival status in the predictive nomogram is shown in scatter plot. (**A**) Risk score ranking of patients. (**B**) Distribution of risk score and associated overall survival.

The patients were divided into two groups according to the scoring of the nomogram (Figure 6A). We utilized a scatter plot to reveal the relationship between the level of risk score and the overall survival of diffuse-type GC. As a result, the high-risk group exhibited significantly poorer overall survival (Figure 6B).

### 3.8. Gene Set Enrichment Analysis (GSEA)

**Figure 7:**
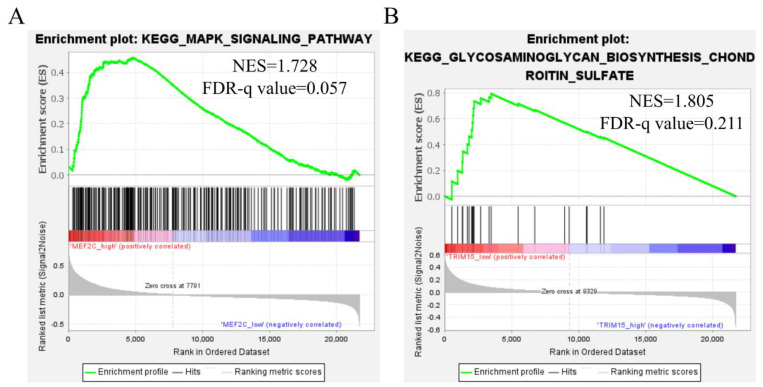
Gene set enrichment analysis was used to showed MEF2C high expression group (**A**) and TRIM15 low expression group (**B**) enriched signaling pathways.

To further investigate the molecular mechanism of MEF2C and TRIM15 in diffuse-type gastric cancer, GSEA was conducted. The results showed high expression MEF2C enriched in the MAPK signaling pathway (Figure 7A) and low expression TRIM15 enriched in glycosaminoglycan biosynthesis chondroitin sulfate (Figure 7B). In addition, the high-risk group was compared to the low-risk group in GSEA. We demonstrated that the high-risk group was significantly enriched in the top 13 biological pathways, including “Regulation of actin cytoskeleton”, “Focal adhesion”, “Vasopressin regulated water reabsorption”, “Dilated cardiomyopathy”, “Glycosaminoglycan biosynthesis chondroitin sulfate”, “Calcium signaling pathway”, “Notch signaling pathway”, “ECM receptor interaction”, “Phosphatidylinositol signaling system”, “MAPK signaling pathway”, ”Hypertrophic cardiomyopathy”, “Vascular smooth muscle contraction”, “FcγR-Mediated Phagocytosis” (Supplementary Materials Figure S1).

## 4. Discussion

Compared to intestinal-type gastric cancer, diffuse-type gastric cancer exhibits a more aggressive phenotype with a relatively poor prognosis and a 5-year overall survival rate of 32.1% [15]. It is widely acknowledged that the treatment failure of diffuse-type gastric cancer is due to drug resistance and disease progression, including tumor recurrence and metastasis. The prognostic model is important to clinicians to provide individualized treatment by determining which patients would benefit most from a particular or a combination of treatment approaches, including radical surgery, adjuvant chemotherapy, neoadjuvant chemotherapy, targeted molecular medicine or immunotherapy. However, today the big problem is that the prognostic model based on clinical characteristics and histopathological characteristics is not accurate [16,17]. Accordingly, it is important to develop a novel prognostic model for improving patient management by stratifying patients according to their characteristics.

In the present study, we constructed a nomogram that incorporated a two-gene (MEF2C and TRIM15) signature and clinicopathological parameters to assist clinicians in determining the prognosis of individual diffuse-type GC patients. The sensitivity and specificity of our prognostic model were more satisfying than the TNM staging system (Figure 5). Gene set enrichment analysis showed that MEF2C and TRIM15 were closely related to invasive and metastasis signaling pathways in diffuse-type gastric cancer. The MAPK signaling pathway was the most significant in the high MEF2C expression group of diffuse-type gastric cancer (Figure 7A). Moreover, the glycosaminoglycan biosynthesis chondroitin sulfate signaling pathway was the most enriched in the low TRIM15 expression group of diffuse-type gastric cancer (Figure 7B). MEF2C upregulation and TRIM15 downregulation in diffuse-type gastric cancer were related to poor prognoses.

In recent years, multiple gene signatures, mRNAs or non-coding RNAs have been used to evaluate the prognosis of gastric cancer patients [18,19,20]. Nevertheless, rare studies have focused on the Lauren subtype-specific gene signature to evaluate the prognosis of diffuse-type gastric cancer. Moreover, few studies have sought to combine the TNM stage with the multi-gene signature to assess the prognosis of diffuse-type gastric cancer. A previous study reported a three-gene signature to predict the prognosis of diffuse-type gastric cancer [21]. However, the prognostic model only considered the three-gene expression level, but lacked the clinical parameters of the diffuse-type gastric cancer patients. The TNM staging system only considers tumor invasion depth, lymph node metastasis and distant metastasis. The biological characteristics of the tumors, such as the immune infiltration status, drug response and intracellular signal pathways are not reflected in the TNM staging system. However, the genomic sequence of the tumor is an effective tool to uncover heterogeneous malignance [22,23]. Several tumor biomarkers can help guide treatment decisions, including Human Epidermal Growth Factor Receptor-2 (HER2), Programmed Cell Death-Ligand 1 (PDL1) and Vascular Endothelial Growth Factor Receptor (VEGFR) [24,25,26]. Accordingly, in the current study, we identified risk factors, including age, T stage, N stage, M stage, the expression level of TRIM15 and MEF2C and established a prognostic model. Finally, a nomogram integrating a two-gene signature and clinicopathologic features was constructed and yielded an accurate prediction of overall survival. Through ROC curves, Kaplan–Meier analysis and decision curve analysis of the external validation in the TCGA-STAD dataset, as a supplement to AJCC staging, our two-gene signature and nomogram demonstrated a similar predictive performance with the training set (Figure 5). Our predictive nomogram will exhibit a potential value of diffuse-type GC in future clinical practice. Similarly, several previous studies integrated clinical features and risk scores based on the expression level of risk genes into a novel prognostic nomogram [27,28,29]. The predictive value of their integrated nomograms was also better than using the risk factor alone. These studies and our present study have a certain reference significance for future clinical research.

MEF2C and TRIM15 have previously been reported to be associated with gastric cancer. Interestingly, Myocyte Enhancer Factor 2C (MEF2C) has been documented in pathways of organelle biogenesis and maintenance and transcriptional misregulation in cancer, which involved DNA-binding transcription factor activity and protein heterodimerization activity. MEF2C has been associated with DNA methylation and enhanced PD-L1 expression in gastric cancer [30,31]. Recent studies have also shown that MEF2C plays an important role in myocilin mediating cancer-induced muscle wasting and cachexia in cancer patients [32] and regulates chemotherapeutic resistance [33] and the disease progression of acute myeloid leukemia [34]. TRIM15 is a member of the tripartite motif (TRIM) family. The protein encoded by TRIM15 has a TRIM motif, including three zinc-binding domains, a RING, a B-box type 1, a B-box type 2 and a coiled-coil region. However, the biological function of TRIM15 remains unknown. Our GSEA results showed that TRIM15 was correlated with the glycosaminoglycan biosynthesis chondroitin sulfate signaling pathway in diffuse-type gastric cancer. Importantly, a recent study has found that the expression of TRIM15 is an independent risk factor of prognosis in gastric cancer patients [35]. However, the roles of the MEF2C and TRIM15 genes in diffuse-type gastric cancer are still unclear. Our current study disclosed that MEF2C and TRIM15 could promote invasion and metastasis through cancer-related signaling pathways: the MEF2C-activated MAPK signaling pathway and the TRIM15-activated glycosaminoglycan biosynthesis chondroitin sulfate signaling pathway (Figure 7). Moreover, our studies revealed poor prognoses associated with upregulated MEF2C and downregulated TRIM15 expression in diffuse-type gastric cancer. Accordingly, the present research revealed the roles of these two genes in diffuse-type gastric cancer and established a risk model to complement the AJCC staging system to improve the outcomes of diffuse-type gastric cancer. However, further in vivo studies are needed to explore the molecular mechanism underlying the oncological function of these two genes in diffuse-type gastric cancer.

This study contains several limitations. First, our study was based on RNA sequence data rather than proteomics, which could have affected the accuracy of our prediction model. Accordingly, the expression of these two genes should be analyzed in another study with a large sample of diffuse-type gastric cancer patients to validate the predictive performance of our model. Furthermore, it may be hard to promote the utilization of multi-genome sequencing during clinical practice due to its high price and practicability. With the development of sequencing technology and precision medicine, the identified two-gene signature will be clinically feasible. Moreover, our predictive model should be externally validated with another large sample of diffuse-type gastric cancer patients.

## 5. Conclusions

In summary, we constructed a nomogram that incorporated a two-gene signature and clinicopathological parameters to assist clinicians in determining the prognosis of individual GC patients. Our nomogram is simple to use and can be harnessed to provide optimal treatment and make medical decisions. To the best of our knowledge, the two-gene prognostic signature described and the nomogram constructed have not been reported previously. The current study provides a new perspective of the molecular mechanisms underlying prognosis prediction in diffuse-type gastric cancer. In addition, MEF2C and TRIM15 were obtained by a pooled analysis of multiple datasets and are accordingly highly reliable. Importantly, these two genes may be potential molecular targets for the treatment of diffuse-type gastric cancer.

## Figures and Tables

**Table 1:** Details of GEO datasets in this study.

Datasets	Platform	Case No.	Sample Size	Submitter	Application
GSE22377	(G-U133_Plus_2) Affymetrix Human Genome U133 Plus 2.0 Array	43	24 intestinal 19 diffuse	Förster S, MDC, Berlin, Germany	Identification of DEGs
GSE38749	(HG-U133_Plus_2) Affymetrix Human Genome U133 Plus 2.0 Array	15	4 intestinal 10 diffuse 1 mixed	Pasini FS, Faculdade de Medicina da USP, São Paulo, Brazil	Identification of DEGs
GSE47007	(HG_U95Av2) Affymetrix Human Genome U95 Version 2 Array	30	18 intestinal 12 diffuse	Sasaki H, National Cancer Center Reseach Institute, Tokyo, Japan	Identification of DEGs
GSE62254	(HG-U133_Plus_2) Affymetrix Human Genome U133 Plus 2.0 Array	300	146 intestinal 134 diffuse 17 mixed 3 indeterminate	Nebozhyn M, Merck, Inc., PA, USA	Validation

**Table 2:** Stratified analysis of COL4A3, MEF2C and TRIM15 for diffuse-type gastric cancer patients (*n* = 134) in terms of prognosis.

Clinical Features		COL4A3 Expression		*p* Value	MEF2C Expression		*p* Value	TRIM15 Expression		*p* Value
		High	Low		High	Low		High	Low	
Age	≤60	32	36	0.151	45	23	0.016 *	31	37	0.014 *
	>60	23	43		30	36		44	22	
Gender	Male	28	32	0.234	38	22	0.122	31	29	0.366
	Female	27	47		37	37		44	30	
Depth of invasion	T2	18	46	0.014 *	25	39	0.001 **	40	24	
	T3	32	28		43	17		31	29	
	T4	5	5		7	3		4	6	
Lymph node	N0	4	4	0.178	5	3	0.33	4	4	0.678
metastasis	N1	16	37		25	28		33	20	
	N2	18	23		27	14		22	19	
	N3	17	15		18	14		16	16	
Distant metastasis	M0	45	69	0.377	60	54	0.063	64	50	0.925
	M1	10	10		15	5		11	9	
AJCC stage	I	1	4	0.59	2	3	0.008 **	4	1	0.51
	II	8	26		11	23		21	13	
	III	23	26		33	16		27	22	
	IV	23	23		29	17		23	23	

* *p* value < 0.05; ** *p* value < 0.01.

**Table 3:** The univariate Cox regression analysis between three genes and other clinical characteristics and OS in diffuse-type gastric cancer.

Characteristics		HR	95% CI	*p* Value
	Age	1.005	0.987–1.023	0.617
Gender	Female	1		
	Male	0.7712	0.495–1.203	0.252
Depth of Invasion	T2	1		
	T3	1.906	1.181–3.078	0.008 **
	T4	2.769	1.301–5.893	0.008 **
Lymph Node of Metastasis	N0	1		
	N1	1.488	0.346–6.398	0.593
	N2	3.177	0.753–13.411	0.115
	N3	11.017	2.608–46.539	0.001 **
Distant Metastasis	M0	1		
	M1	3.677	2.172–6.224	<0.0001 ***
Gene Expression	COL4A3 expression	1.281	0.685–2.395	0.438
	MEF2C expression	3.97	1.512–10.430	0.005 **
	TRIM15 expression	0.2861	0.104–0.790	0.015 *

* *p* value < 0.05; ** *p* value < 0.01; *** *p* value < 0.001; HR, hazard ratio; CI, confidence interval.

## Data Availability

All dataset analysis for this study can be found in the Gene Expression Omnibus (GEO) database, the Cancer Genome Atlas (TCGA) database and the website of Kaplan–Meier Plotter.

## Supplementary Materials

The following supporting information can be downloaded at: https://www.mdpi.com/article/10.3390/curroncol30010014/s1, Figure S1: GSEA showing the top 13 enriched biological pathways of high-risk group.
