# Supplementary figures and images for: Identification of a Two-Gene Signature and Establishment of a Prognostic Nomogram Predicting Overall Survival in Diffuse-Type Gastric Cancer
Source: Curr Oncol. 2022 Dec 23;30(1):171–83. doi: 10.3390/curroncol30010014 (PMC9857582; doi:10.3390/curroncol30010014)

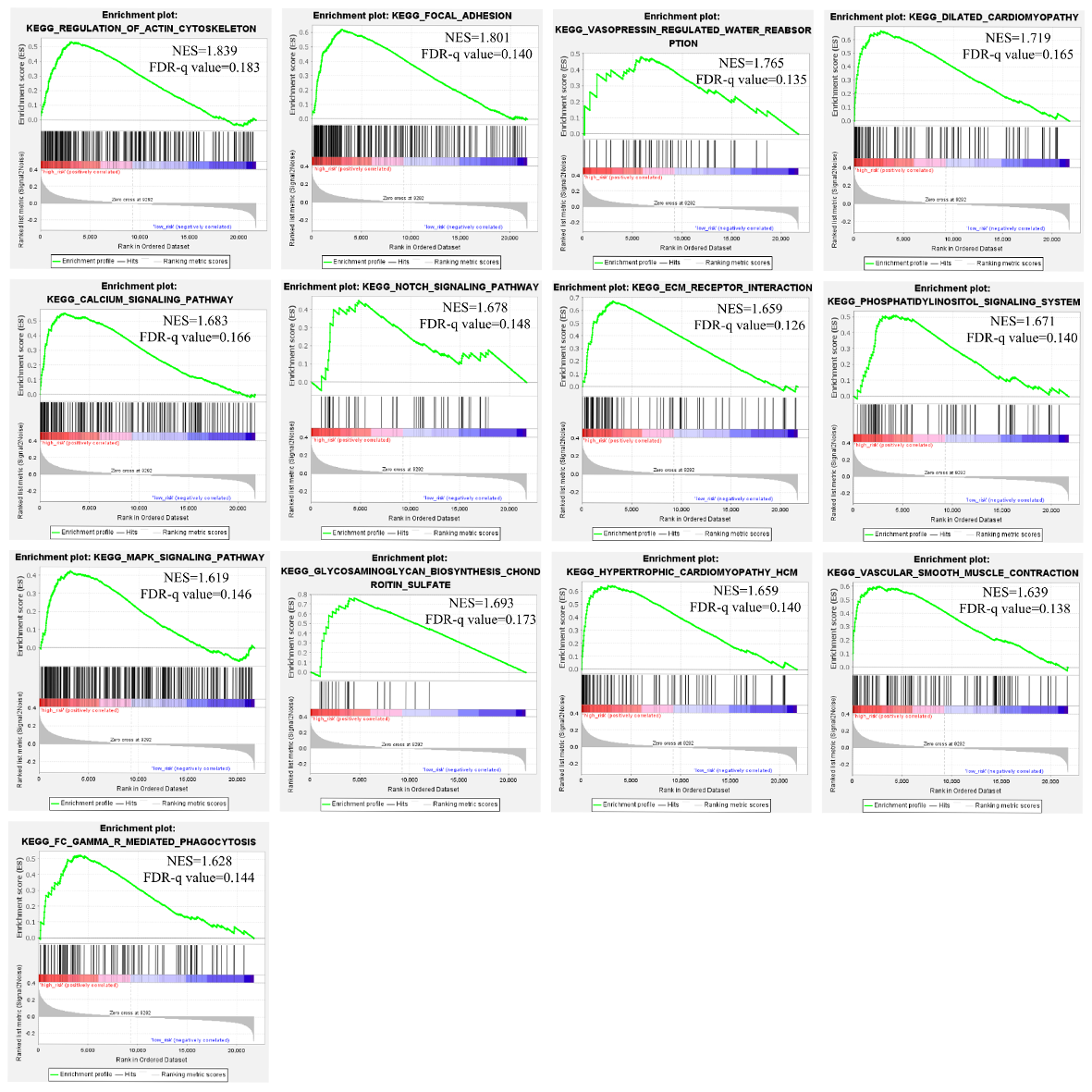

Supplement: Supplementary file 1 [file curroncol-30-00014-s001.zip › curroncol-2093318-supplementary.tif]
